# Atopic dermatitis and autoimmunity: the occurrence of autoantibodies and their association with disease severity

**DOI:** 10.1007/s00403-019-01890-4

**Published:** 2019-02-23

**Authors:** James Holmes, Lucy C. Fairclough, Ian Todd

**Affiliations:** School of Life Sciences, University of Nottingham, Life Sciences Building, University Park, Nottingham, NG7 2RD UK

**Keywords:** Atopic dermatitis, Autoimmunity, Autoantibody, IgE, IgG, IgA, Anti-nuclear autoantibodies

## Abstract

Atopic dermatitis (AD) is a widespread condition that appears to be increasing in prevalence and severity worldwide, yet the underlying mechanisms are not well understood. Recent research has identified various similarities between AD and autoimmune conditions, as well as indicating that there may be an association between AD and autoimmunity. This systematic review evaluates the association between AD and autoimmunity, as well as between severity of disease in AD and autoimmunity, with an emphasis on the associations with autoantibodies. MEDLINE (1946 to December 2017) and Embase (1974 to December 2017) databases were searched. Further relevant articles were retrieved from reference lists. Only studies measuring direct indicators of autoimmunity, in humans, were included. Qualitative analysis was carried out for all studies. In addition, quantitative analysis was used to evaluate prevalence of IgE autoantibodies and anti-nuclear antibodies (ANAs) in AD patients and control subjects. The Mantel–Haenszel method was used with a random-effects model. 28 studies assessed the occurrence of autoantibodies in AD patients and 16 studies were used to evaluate association between disease severity and autoantibodies. Pooled analysis from 14 studies, involving 986 AD patients and 441 control subjects, showed that IgE autoantibodies were significantly more prevalent in patients with AD (*P* < 0.00001) than control subjects. Similar analysis was carried out for ANAs, with eight studies that involved 1045 AD patients and 1273 control subjects. ANAs were significantly more prevalent in patients with AD (*P* = 0.003). This quantitative analysis supported an association between AD and IgE autoantibodies, as well as between AD and ANAs. There was insufficient data to make similar conclusions for other indicators of autoimmunity. The weight of evidence also suggests an association between IgE autoantibodies and disease severity. There was insufficient evidence to make this link for other indicators of autoimmunity.

## Introduction

The term Atopic Dermatitis (AD) was first used by Wise and Sulzberger in 1933 [1]. The World Allergy Organisation defines eczema (also known as atopic eczema dermatitis syndrome (AEDS) and as AD throughout this review) as ‘an inflammatory, chronically relapsing, non-contagious and extremely pruritic skin disease’ [2].

Prevalence of AD worldwide is high, with 7.9% of 6–7 years presenting with the condition in the ISAAC Phase 3 study [3]. AD appears to be increasing in prevalence globally, with centres reporting increases in self-reported AD and severe AD [3]. There was significant variance in the prevalence of AD between centres, both within and across regions, which has been attributed, in part, to environmental differences [4]. The increasing prevalence and severity of AD makes it essential to understand the underlying mechanisms of the disease, enabling more effective future treatments.

AD is traditionally thought of as an allergic condition with extrinsic allergens driving the immune response. The discovery of a deficiency in the stratum corneum caused by filaggrin gene mutations supports this, as impaired skin barrier function allows for allergens to infiltrate the body, inducing an immune response [5]. AD patients with these mutations are more likely to have persistent, severe, allergen-driven symptoms, but some patients continue to suffer from AD despite removal of stimuli such as dust mites or pollen [6]. Overall, as many as two-thirds of AD patients demonstrate ‘no measurable allergen-specific IgE antibody sensitization’ [7].

One hypothesis as to why chronic inflammation persists, despite removal of environmental allergens, is infection by *Staphylococcus aureus. S. aureus* colonization was found in more than 90% of patients and was found to correlate significantly with severity [8]. *S. aureus* has a contribution to most AD lesions, both by worsening inflammation through direct action of toxins and by increasing production of IgE antibodies [9]. It would follow that other skin based microorganisms, such as the fungus *Malassezia sympodialis*, could also cause this type of exacerbation, but this does not account for patients that suffer AD lesions without allergic sensitisation.

Despite the traditional understanding of AD, there has been evidence to suggest there might be an autoimmune component to the disease. Disease progression in AD is similar to that in known autoimmune conditions, involving periods of relapse and remission. Furthermore, a significant association has been found between AD and 11 autoimmune conditions, as well as the fact that AD patients are more likely to present with ‘multiple autoimmune comorbidities’ [10]. Finally, immediate skin reactions to some human proteins indicate that, for some patients, IgE autoantibodies are produced [11] which could contribute to disease progression. Researchers have also recently claimed that they have proven that ‘atopic dermatitis … is an immune-driven (autoimmune) disease’ [12] by treating AD with Dupilumab. This blocked IL-4 and IL-13 [13], to prevent Th2 cytokines from incorrectly targeting autologous tissues.

## Methods

This systematic review investigates the link between AD and autoimmunity (with an emphasis on autoantibodies), providing an update to, and widening of scope of, a systematic review from 6 years earlier [14]. The present review evaluates the strength of the link between AD and autoantibodies, and whether there is an association between AD severity and autoantibodies.

## Identification of relevant studies

Two modifications were made to the search strategy employed in 2012 by Tang et al. [14]: ‘autologous’ was added as a search term and the Embase database was searched in addition to MEDLINE. These alterations ensured the search strategy reflected current terminology and that as many relevant papers as possible were found.

The search terms were as follows:

1. exp Dermatitis, Atopic/
2. atopic dermatitis.mp.
3. dermatitis atopic.mp.
4. exp Eczema/
5. childhood eczema.mp.
6. infantile eczema.mp.
7. 1 or 2 or 3 or 4 or 5 or 6
8. exp Autoimmunity/
9. exp Autoantigens/
10. Autoallerg$.mp.
11. autoreactivity.mp.
12. Profilins/
13. ribosomal P2.mp.
14. exp Thioredoxins/
15. exp immunoglobulin G/
16. exp immunoglobulin E/
17. superoxide Dismutase/
18. autoantibod$.mp.
19. self-antigen.mp.
20. self-react$.mp.
21. autologous.mp.
22. 8 or 9 or 10 or 11 or 12 or 13 or 14 or 15 or 16 or 17 or 18 or 19 or 20 or 21
23. 7 and 22
24. limit 23 to (male and female and humans and humans)

Database searching was carried out during October to December 2017.

**Fig. 1:**
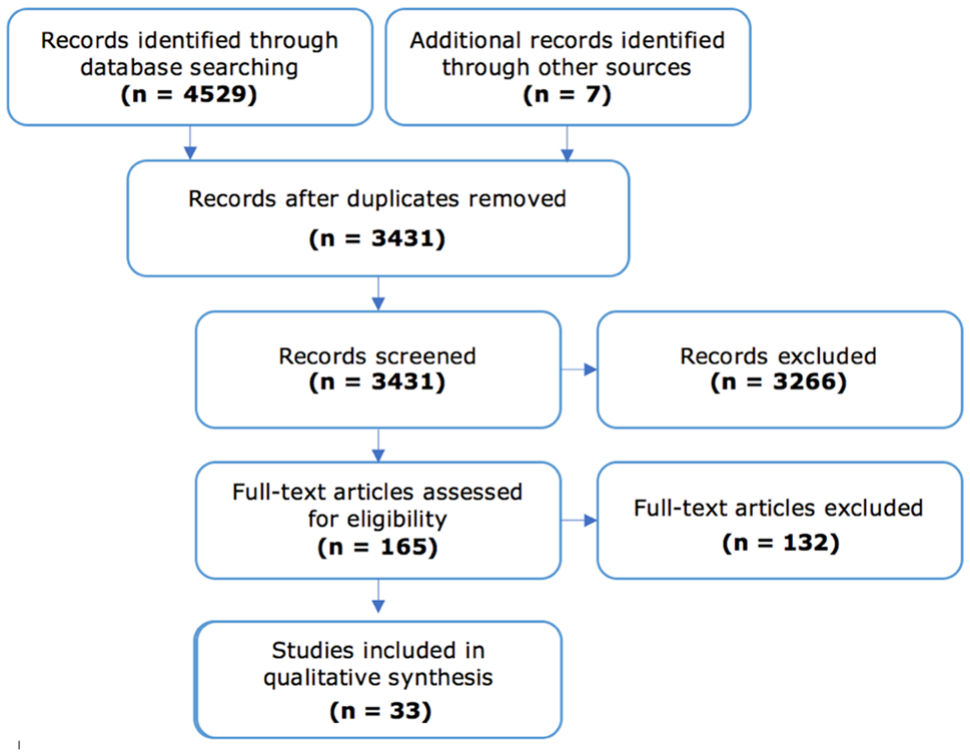
PRISMA diagram (adapted from [16])

The PRISMA diagram [15] in Fig. 1 gives an overview of the number of papers at each stage of the literature search. Titles and abstracts from this search were checked, yielding 165 papers that warranted full-text analysis. These were scanned in full, with 28 studies providing evidence relating to an association between AD and autoantibodies, and 16 that evaluated severity of AD in relation with the occurrence of autoantibodies. During this full-text analysis, the reference sections of papers were checked to identify relevant papers that had not appeared in the search results. Most papers excluded at this point were conference proceedings or included results not linked to autoimmunity and AD.

## Inclusion criteria

### Language

All literatures evaluated in this systematic review were in English (including papers that had already been translated in to English).

### Types of studies

Due to the wide range of study designs present in the literature, all the types of study were included. The main types of study evaluated were case–control studies, case series, and individual case reports.

### Types of participants

This systematic review evaluated all studies involving human participants, irrespective of age or gender. Studies varied in the age ranges of the participants (details in Tables 1, 3, 4, 5, 7): most included adults only or a mixture of children and adults, whereas a few studies included children only. However, there was no apparent effect of participants’ age on the relationship between autoreactivity and AD (Table 2) or severity of AD (Table 6). All the literature was included regardless of whether they had clear criteria for selecting patients with AD. These criteria have been noted for all studies in appendices, but have only been referred to in the main text when these criteria are unclear or missing.

**Table 1:** Studies examining the association of autoantibodies of IgE class with atopic dermatitis

References	Study design and size	Diagnostic criteria for AD	Methods	Prevalence of AR in control subjects	Prevalence of AR in patients with AD
Aichberger et al. [17]	Case–control study 12 patients with eczema 11 patients with rhinoconjunctivitis 6 healthy controls 6 control subjects with chronic dermatoses (All adults. Patients with AD ranged from 20 to 72 years, with a mean age of 38.4. Control subjects had a mean age of 38.6. Insufficient data to calculate SD)	*Clear* Hanifin and Rajka	*Western Blot* Serum IgE against epithelial cell line A431 and rHom s 4	*0%* No control subjects produced serum IgE against epithelial cells (A431) or Hom s 4	*91.7%* 11/12 patients produced serum IgE against epithelial cells (A431) *16.7%* 2/12 patients produced serum IgE against Hom s 4
Altrichter et al. [18]	*Case series* 192 patients with eczema 5 healthy control subjects (All adults. AD patients ranged from 18 to 80 years, with a mean age of 39. Control subjects ranged from 25 to 64 years, with a mean age of 31. Insufficient data to calculate SD)	*Clear* Hanifin and Rajka	*Western blot* Serum IgE against epithelial cell line A431 + epidermis	*0%* 0/26 control subjects displayed any autoreactivity	*28%* 54/192 eczema patients displayed autoreactivity against A431 and/or epidermis
Guarneri et al. [19]	Case–control study 27 patients with AD 27 control subjects (10 children and 17 adults. All participants ranged from 5 to 49 years. AD patients and controls had a mean age of 22.2 ± 12.2)	*Clear* Hanifin and Rajka	*Skin prick test* against hMnSOD	*0%* 0/27 control patients	*14.8%* 4/27 patients
Hiragun et al. [20] IgE and IgG	Case–control study 63 patients with AD 24 patients with Cholinergic Urticaria 32 patients with bronchial asthma 14 patients with allergic rhinitis 23 normal control patients (Patients with AD ranged from 0 to 65 years, with a mean age of 29.9 ± 11.5. Other participants in this study varied from 0 to 82)	*Moderately Clear* ‘severity of AD was evaluated using severity index of Japanese guideline for AD’	*ELISA* Specific to QRX-specific IgE and rMGL_1304-specifc IgE	*26.1%* 6/23 controls were QRX-specific IgE positive *39.1%* 9/23 controls were rMGL_1304-specific IgE Positive	*66.7%* 42/63 AD patients were QRX-specific IgE positive (*P* = 0.0012) *61.9%* 39/63 AD patients were rMGL_1304-specific IgE Positive (*P* = 0.0857)
Ilves, Virolainen and Harvima [21]	Case–control study 50 patients with AD 24 control subjects (All adults. Patients with AD ranged from 21 to 64 years, with a mean age of 38. Control subjects ranged from 21 to 68 years, with a mean age of 42. Insufficient data to calculate SD)	Clear Rajka and Langeland	*Intracutaneous injection and ImmunoCAP* against autologous sweat	*50%* 1/24 positive 11/24 weakly positive 12/24 negative	*72%* 19/50 positive 17/50 weakly positive 14/50 negative *(difference P* < *0.008)*
Kawamoto et al. [22] IgE and IgG	Case–control study 40 patients with AD 26 control patients (no clinical history of allergic diseases and serum IgE within normal levels) (Patients with AD ranged from 12 to 44 years, with a mean age of 26.4. Control subjects ranged from 19 to 54 years, with a mean age of 29.2 No SD provided and insufficient data to calculate)	*Clear* UK Working Party’s Diagnostic Criteria for Atopic Dermatitis	*ELISA* Peptide Specific	–	*No significant difference* in IgG levels or number of positive samples *for 5 peptides* *Significant difference* in both IgG levels and number of positive samples for *SART*_*2161*_, *SART*_*2899*_, *and ART4*_*13*_
Kortekangas-Savolainen et al. [23]	Case–control study 27 patients with IgE-mediated AEDS 13 control subjects (6 healthy controls, 4 patients with urticaria, 3 patients with psoriasis) (All adults. Patients with AD had a mean age of 33 ± 11. Control subjects had a mean age of 47 but insufficient data to calculate SD.)	*Not clear*	*Western blot* Serum IgE against keratinocytes	*0%* 0/13 control subjects displayed IgE autoantibodies against keratinocytes	*37%* 10/27 patients displayed IgE autoantibodies against keratinocytes
Mittermann et al. [24]	Case–control study 11 patients with AD 7 patients with rhinoconjuntivitus 5 patients with contact allergy 9 non-atopic individuals (All adults, except 1 child with AD. Patients with AD ranged from 13 to 59 years, with a mean of 31.9 ± 14.9. Control subjects ranged from 26 to 63 years, with a mean of 35.2 ± 10.9.)	*Clear* Hanifin and Rajka	*Immunoblotting* Against human epithelial cell extracts	*0%* 0/12 control subjects	*72.7%* 8/11 patients
Mittermann *et al*. (2016) [25]	Case–control study 179 patients with AD (53 severe and 126 moderate) 140 patient control group (43 patients with seborrheic eczema and 97 individuals with no previous allergies or skin conditions) (All adults. All participants ranged from 18 to 65 years. AD patients had a mean age of 28, control subjects had a mean age of 36. No SD provided and insufficient data to calculate)	*Clear* UK Working Party’s Diagnostic Criteria for Atopic Dermatitis	*Immunoblotting* Against human A431 extract	*0%* 0/140 control subjects	*18%* 32/179 patients with AD
Mothes et al. [26]	Case–control study 174 patients with eczema 10 patients with psoriasis 26 healthy controls (All adults. Eczema patients had a mean age of 35.4 ± 14.8. Control subjects had a mean age of 36.5 ± 16.5.)	*Clear* Hanifin and Rajka	*Immunoblotting* IgE reactivity to a variety of human epithelial antigens	*0%* 0/10 patients with psoriasis and 0/26 healthy controls displayed autoreactivity	*23%* 40/174 patients displayed autoreactivity
Natter et al. [27]	*Case series* 51 patients with eczema (14 children and 37 adults. Patients with eczema ranged from 1 to 63 years, with a mean age of 26.9 ± 18.6)	*Clear* Hanifin and Rajka	*Western Blot* Whole serum IgE against endothelial cells and A431	–	*43.1%* 22/51 patients
Schmid-Grendelmeier *et al*. (2005) [28]	Case–control study 69 patients with eczema 5 healthy controls13 patients with psoriasis 11 patients with ABPA 13 patients with allergy to A fumigatus (All adults. Eczema patients had a mean age of 29.3 ± 5.6. Other participants had a mean age of 25.3, but insufficient data to calculate SD)	*Clear* Hanifin and Rajka	*ELISA and SPT* against rhMnSOD	*100%* 11/11 patients with ABPA *0%* 0/31 other control subjects	*42%* 29/69 patients with eczema
Valenta et al. [29]	Case–control study 20 patients with AD 28 control patients including patients with various allergic conditions and non-allergic healthy individuals (Patients with AD ranged from 17 to 52 years with a mean age of 35.3 ± 12.3. Control subjects ranged from 18 to 64 years with a mean age of 33.0 ± 11.4)	*Clear* Hanifin and Rajka	*Western Blot* IgE antibodies against endothelial cells, platelets, fibroblasts and epithelial cells	*0%* 0/28 tested positive for IgE against human proteins from most cell extracts	*60%* 12/20 tested positive for IgE against human proteins from most cell extracts
Zeller et al. [30] IgG, IgA, IgE	Case–control study 71 patients with AE 12 patients with psoriasis 24 healthy control subjects (Patients with AE had a mean age of 33.35 ± 12.70. Patients with non-atopic AE had a mean age of 39.35 ± 13.5. Patients with psoriasis had a mean age of 45.17 ± 9.20. Healthy control subjects had a mean age of 29.00 ± 3.71)	*Clear* Hanifin and Rajka and European Academy of Allergology and Clinical Immunology	*Immunoblotting and ELISA* Specific to actin-α, eIF6, RP1, HLA-DR-α and tubulin-α	*0%* 0/36 control patients	*71.8%* 51/71 of all patients with Atopic Eczema *72.2%* 13/18 patients with the non-atopic form of AD

**Table 2:** Association of autoantibodies with atopic dermatitis

Marker of autoimmunity	Papers that indicate autoreactivity is more common in patients with AD	Papers that do not indicate autoreactivity is more common in patients with AD
IgE autoantibodies	**13** Aichberger et al. [17] Altrichter et al. [18] Guarneri et al. [19] Hiragun et al. (for QRX) [20] Ilves, Virolainen and Harvima [21]* Kortekangas-Savolainen et al. [23] Mittermann et al. [24, 25] Mothes et al. [26] Natter et al. [27] Schmid-Grendelmeier et al. [28] Valenta et al. [29] Zeller et al. [30]	**2** Hiragun et al. (for rMGL_1304) [20]^**†**^ Kawamoto et al. [22]^§^
IgG autoantibodies	**4** Bergman et al. [31]* (for CCL-3) Neuber et al. [32]* Ochs et al. [33] Szakos et al. [34]*	**5** Ambrozic et al. [35] Bergman et al. [31] (*for 5 autoantigens*)^**†**^ Du Toit et al. [16] El-Rachkidy et al. [36] Kawamoto et al. [22] *(for* 5 peptides)**, *(for* 3 *peptides)*^*§*^
IgA autoantibodies	**0**	**1** Ress et al. [37]
ANAs	**7** Dhar, Kanwar and Deodhar [38] Higashi et al. [39] Ochs et al. [33] Ohkouchi et al. [40]* Szakos et al. [34] Tada et al. [41] Taniguchi et al. [42]	**1** Ress et al. [43]^**†**^

*Differences were statistically significant

**†**Differences were not statistically significant

^§^Autoreactivity was significantly lower in AD patients

**Table 3:** Studies examining the association of autoantibodies of IgG class with atopic dermatitis

References	Study design and size	Diagnostic criteria for AD	Methods	Prevalence of AR in control subjects	Prevalence of AR in patients with AD
Ambrozic et al. [35]	Case–control study 45 patients with AD 26 patients with other atopic conditions (controls) (All children. Patients with AD ranged from 2 months to 16.8 years, with a mean age of 3.7. Control subjects ranged from 2.1 to 17.8 years, with a mean age of 11.0. Insufficient data to calculate SD)	*Moderately Clear* ‘Sera selected from the serum banks of the Pediatrics Clinic and Department of Rheumatology (University Medical Centre, Ljubljana)’	*ELISA* Specific to Anti-β2GPI	*38%* 10/26 control patients	*42%* 19/45 patients with AD *No significant difference*
Bergman et al. [31]	Case–control study 37 Patients with Psoriasis 18 patients with AD 56 healthy controls (All adults. Patients with psoriasis ranged from 20 to 76 years, with a median age of 43.5. Patients with AD ranged from 19 to 69 years, with a median age of 30. Healthy subjects ranged from 27 to 62 years, with a median age of 45.5)	*Clear* Hanifin and Rajka	*ELISA* Against a variety of autoantigens	Mean Log2Ab Titer of autoantibody (HCs) TNF-α: 7.61 IFN-α: 7.43 CCL-5: 7.48 CCL-2: 7.84 CCL-3: 7.59 IL-17: 7.41	Mean Log2Ab Titer of autoantibody TNF-α: 7.94 IFN-α: 7.76 CCL-5: 7.47 CCL-2: 7.41 CCL-3: 8.71 (*P* = 0.035) IL-17: 7.59
Du Toit *et al*. (2006) [16]	Case–control study 33 patients with AEDS 78 patients with Chronic Urticaria (All children. Patients with AEDS ranged from 1.25 to 19 years, with a mean age of 7.6. Patients with Chronic Urticaria were age matched)	*Not clear*	*Anti–Fcε receptor assays* Specific to IgG FcεR1α autoantibodies	*47%* 37/78 patients with CU	*0%* 0/33 patients with AEDS
El-Rachkidy et al. [36]	Case–control study Patients with psoriasis (number varies based on test) 5 pooled patients with AD (Age of patients with AD not specified. Psoriasis patients ranged from 22 to 66 years).	*Not clear*	*Immunoblotting and ELISA* Specific to stratum corneum antigens	Psoriasis patients *were* autoreactive against stratum corneum antigens	AD patients *were not* autoreactive against stratum corneum antigens
Kawamoto et al. [22] (IgE and IgG)	Case–control study 40 patients with AD 26 control patients (no clinical history of allergic diseases and serum IgE within normal levels) (Patients with AD ranged from 12 to 44 years, with a mean age of 26.4. Control subjects ranged from 19 to 54 years, with a mean age of 29.2). Insufficient data to calculate SD	*Clear* UK Working Party’s Diagnostic Criteria for Atopic Dermatitis	*ELISA* Peptide Specific	–	*No significant difference* in IgG levels or number of positive samples *for 5 peptides* *Significant difference* in both IgG levels and number of positive samples for *SART*_*2161*_, *SART*_*2899*_, *and ART4*_*13*_
Neuber et al. 32]	Case–control study 16 patients with AD 72 healthy control subjects (All adults. Patients with anti-CD28 autoantibodies had a mean age of 52.3 ± 18.8, patients without anti-CD28 autoantibodies were had a mean age of 58.7 ± 19.9)	*Moderately clear* Treated at the Department of Dermatology, University Hospital Eppendorf, Hamburg	*ELISA* Specific to Anti-CD28	*11.1%* 8/72 control patients	*87.5%* 14/16 AD patients *(P* < 0.0001*)*
Ochs et al. [33] ANA and IgG	Case–control study 64 patients with eczema 39 control patients (study also included a range of other atopic conditions) (Patients with eczema ranged from 4 to 43 years, with a mean age of 24.4. No ages provided for control subjects. Insufficient data to calculate SD)	*Clear* Hanifin and Rajka	*Indirect immunofluorescence* For ANAs *Western blot* Against anti-DFS70 IgG	*ANA not stated* *WB-DFS70: 0%* 0/39 healthy control subjects	*ANA: 41%* 26/64 patients *WB-DFS70: 29.6%* 19/64 patients
Szakos et al. [34] IgG and IgM (including ANA)	Case–control study 72 patients with AEDS 22 healthy control patients (All children. Patients with AEDS ranged from 2 to 17 years, with a mean age of 8 years. Control subjects ranged from 1.5 to 14 years, with a mean age of 8.6 years. Insufficient data to calculate SD)	*Not Clear*	*Indirect immunofluorescence* For ANAs *ELISA* Specific to APLs	*9.1%* 2/22 healthy control patients had non-allergen-specific IgG and/or IgM antibodies *9.1%* 2/22 healthy control patients displayed ANAs	*29.3%* 21/72 patients had non-allergen-specific IgG and/or IgM antibodies *13.9%* 10/72 AD patients displayed ANAs *APL antibodies occurred significantly more often in AD patients*

### Types of publication

In this systematic review, all papers published in journals were included, with the exception of reviews and editorials. Letters to journals were included, as they were a key source of smaller studies. Grey literature was not included.

### Outcome measures

The papers evaluated in the systematic review were heterogeneous in both study design and outcome measures. The only restriction was that studies had to provide direct evidence of autoantibodies. When evaluating the association between autoantibodies and AD, any tests that indicated autoantibodies, for example, ELISA and skin prick tests (SPTs), were included. When evaluating the association between severity of AD and autoantibodies, all measures of severity were included. The most common measures of severity were SCORAD (SCORing Atopic Dermatitis) [44] and Rajka and Langeland [45] systems, although some authors used other assessments of severity.

### Statistical analysis

Where appropriate, a quantitative analysis was carried out using Review Manager 5.3 [46]. The Mantel–Haenszel method was used with a random-effects model to account for the variation in study design and resultant difference in effect size.

## Results

### Association of autoantibodies with atopic dermatitis

28 papers were identified that fulfilled the inclusion criteria and were relevant to this part of the review. The main indicators of autoimmunity investigated were specific IgE and IgG antibodies, as well as IgG ANAs.

### Autoantibodies of IgE class

**Fig. 2:**
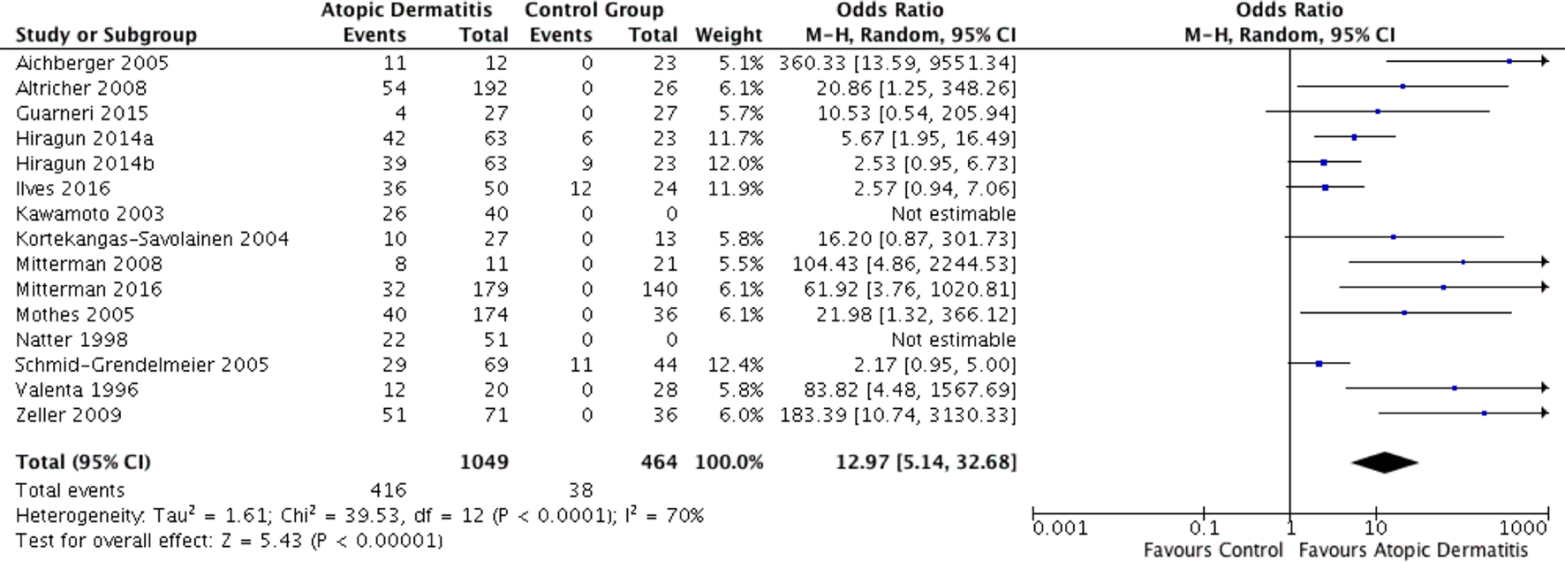
Forest plot showing data from all studies into IgE antibodies. ‘Events’ are instances of autoimmunity, and ‘total’ refers to the total number of people in that group of a study

14 studies investigated the presence of specific IgE autoantibodies in patients with AD; the details of these studies are summarised in Table 1 (in alphabetical order of the first authors). Twelve were case–control studies, of which 11 indicated that certain IgE autoantibodies were significantly more common in patients with AD. The study quality was generally good, with well-described protocols, often using western blot, ELISA or SPT techniques, and robustly defined criteria for selection of AD patients in most cases. Most studies evaluated the presence of IgE autoantigens to epithelial cells, epithelial cell extracts, and other proposed autoantigens associated with the skin. Furthermore, the control groups were comprised predominantly of healthy individuals which makes comparison between, and pooling of, individual studies much easier and more useful. This meant an analysis could be carried out on the combined data, which established an odds ratio of 12.97 (with 95% confidence limits of 5.14–32.68) and *P* < 0.00001, indicating that IgE autoantibodies are significantly more likely to be identified in patients with AD compared to the control groups (Fig. 2). This effect seemed to be uniform across all tested antigens, although the majority of evidence concerns specific antibodies to epithelial cell extracts. A qualitative analysis of the studies also indicated that all but one (13/14) concluded that IgE class autoantibodies are more common in AD patients than controls (Table 2).

### Autoantibodies of IgG class

Eight studies investigated the presence of specific IgG autoantibodies in AD (Table 3). However, it was not possible to carry out a quantitative analysis: whilst the studies predominantly used ELISA techniques, the study design was too varied for a pooled analysis. Some studies only compared AD patients against patients with other conditions, for example psoriasis, whilst others only provided mean titres. Furthermore, only three of the studies had clear definitions for AD diagnosis. Because of the more variable study design, it was more appropriate to carry out a qualitative review. Levels of IgG autoantibodies specific to SART2_161_, SART2_899_, ART4_13_ [22], CCL3 [31] and CD28 [32], as well as APL antibodies [34] were significantly higher than in their respective control groups, but levels of autoantibodies specific to β_2_GPI [35], TNF-α, IFN-α, CCL-5, CCL-2, IL-17 [31], and five other peptides [22] were not significantly higher. Autoantibodies against DFS70 [33] and Fc_ε_R1_α_ autoantibodies [16] were more commonly identified in AD patients than the respective control groups, but no statistical analysis was carried out. Finally, AD patients were found not to display an autoreactive response to stratum corneum antigens, but psoriasis patients did (although no statistical analysis was carried out). Overall, there is no clear picture as to the presence of IgG autoantibodies in AD patients (Table 2). The mixed results, combined with more variable study techniques and less clarity with respect to inclusion criteria, means that more research is required into the relationship between AD and IgG autoantibodies.

### Autoantibodies of IgA class

Only one study solely investigated IgA autoantibodies in AD: Ress et al. [37] carried out a large case–control study, comprising 297 patients with AD and 52 healthy control subjects. ELISA techniques were used to determine the presence of IgA-anti-transglutaminase 1 (anti-TG1) and IgA-anti-transglutaminase 3 (anti-TG3) in the control group and AD group. However, this study did not indicate an association between IgA autoantibodies and AD (Table 2), but there are insufficient studies to draw a clear conclusion.

### Anti-nuclear autoantibodies

**Fig. 3:**
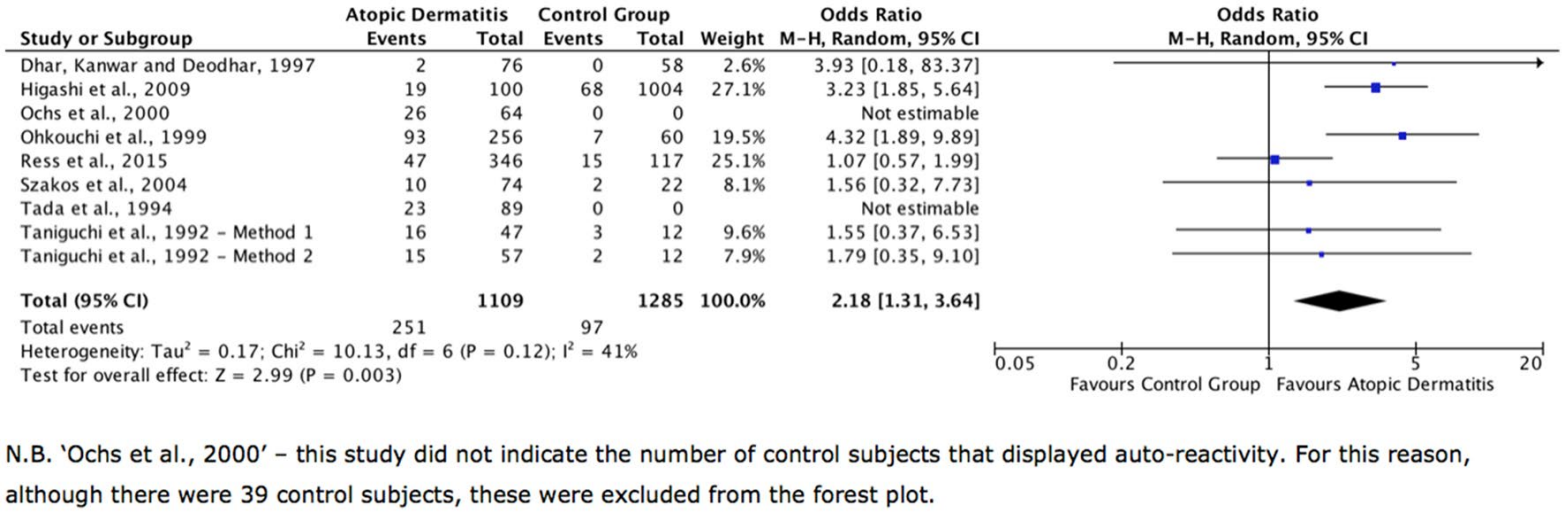
Forest plot showing data from all studies into ANAs. ‘Events’ are instances of autoimmunity, ‘Total’ refers to the total number of people in that group of a study

Eight studies investigated ANAs in AD (Table 4), with two of these including ANAs as part of a wider study. Six of the eight were case–control studies. Most of these papers applied clear, commonly used definitions of AD and most used well-described methods—particularly indirect immunofluorescence—to identify ANAs. Seven of the eight papers indicate that ANAs occurred more commonly in patients with AD than in the control subjects (Table 2). Furthermore, the pooling of data showed that patients with AD have a significantly higher frequency of ANAs than the control subjects with an odds ratio of 2.18 (95% confidence intervals 1.31–3.64) and *P* = 0.003 (Fig. 3).

**Table 4:** Studies examining the association of anti-nuclear autoantibodies with atopic dermatitis

References	Study design and size	Diagnostic criteria for AD	Methods	Prevalence of AR in control subjects	Prevalence of AR in patients with AD
Dhar et al. [38]	Case–control study 76 children with AD 58 age matched control subjects (All children. Patients with AD ranged from 6 months – 12 years, with a mean age of 3.4 years. Control subjects were age matched. Insufficient data to calculate SD.)	*Clear* Hanifin and Rajka	*FITC-conjugated goat antihumag [sic] IgG*	*0.0%* 0/58 control subjects displayed ANAs at a titre of 1:40	*2.6%* 2/76 patients with AD displayed ANAs at a titre of 1:40
Higashi et al. [39]	Case–control study 100 patients with AD 1004 control subjects (Patients with AD ranged from 2 to 64 years, with a mean age of 28.2 years. Control subjects ranged from 20 to 70 years, with a mean age of 48.8 years. Insufficient data to calculate SD.)	*Clear* Hanifin and Rajka	*Indirect immunofluorescence* Against ANA	*6.8%* 68/1004 control patients (between titres of 40X–640X)	*19%* 19/100 patients with AD (between titres of 40X–640X)
Ochs et al. [33] ANA and IgG	Case–control study 64 patients with eczema 39 control patients (study also included a range of other atopic conditions) (Patients with eczema ranged from 4 to 43 years, with a mean age of 24.4 years. Insufficient data to calculate SD. No ages provided for control subjects.)	*Clear* Hanifin and Rajka	*Indirect immunofluorescence* Against ANA	*Not stated*	*41%* 26/64 patients
Ohkouchi et al. [40]	Case–control study 256 patients with eczema 60 control subjects (All adults. Patients with eczema had a mean age of 23 years. Control subjects were age matched. Insufficient data to calculate SD.)	*Clear* Hanifin and Rajka	*Indirect immunofluorescence* Against ANA	*11.7%* 7/60 control patients at a 40 fold dilution	*36.3%* 93/256 AD patients at a 40 fold dilution (*P* < 0.001)
Ress *et al*. (2015) [43]	*Cross-sectional study* 346 children with active AD 117 hospital controls without known skin diseases, at similar ages (All children. Patients with AD ranged from 0.5 to 18.8 years, with a mean age of 5.8 years. Control subjects ranged from 0.5 to 17.7 years, with a mean age of 7.9 years. Insufficient data to calculate SD.)	*Clear* UK Working Party’s Diagnostic Criteria for Atopic Dermatitis	*Indirect immunofluorescence* Against ANA	*12.8%* 15/117 control patients were ANA Positive at a titre of 1:10	*13.6%* 47/346 patients with AD were ANA Positive at a titre of 1:10 (*P* > 0.05)
Szakos et al. [34] IgG and IgM (including ANA)	Case–control study 72 patients with AEDS 22 healthy control subjects (All children. Patients with AEDS ranged from 2 to 17 years, with a mean age of 8.0 years. Control subjects ranged from 1.5 to 14 years, with a mean age of 8.6 years. Insufficient data to calculate SD.)	*Not clear*	*Indirect immunofluorescence* Against ANA	*9.1%* 2/22 healthy control patients displayed ANAs	*13.9%* 10/72 AD patients displayed ANAs
Tada et al. [41]	*Case series* 89 patients with AD (Patients with AD ranged from 5 to 49 years, with a mean age of 19.0 ± 7.4 years)	*Clear* Hanifin and Rajka	*Indirect immunofluorescence* Against ANA of the IgG subclass	-	*25.8%* 23/89 displayed ANAs at dilutions of 1:40–1:640
Taniguchi et al. [42]	Case–control study 47 AD patient sera analysed using method 1 57 AD patient sera analysed using method 2 12 control subject sera analysed using methods 1 and 2 (all adults) All adults. Patients with AD ranged from 15 to 30 years. Insufficient data to calculate mean and SD. No data provided on control subjects	*Clear* Hanifin and Rajka	*FITC-conjugated polyvalent immunoglobulins* *(method 1)* *FITC-conjugated anti-IgG* *(method 2)*	*25%* 3/12 healthy control subjects had ANAs identified using method 1 *16.7%* 2/12 healthy control subjects had ANAs identified using method 2	*34.0%* 16/47 AD patients with ANA identified using method 1 *26.3%* 15/57 AD patients with ANA identified using method 2

## Association of autoantibodies with severity of atopic dermatitis

The second part of this systematic review considers whether there is an association between severity of disease in patients with AD and the presence of an autoimmune response. From the papers identified using the search strategy, 16 papers evaluated the presence of autoimmunity in combination with disease severity. 13 papers provide evidence of the relationship between IgE autoantibodies and disease severity (Tables 5, 6), and two provide research relating to ANAs (Table 6). The final paper investigated ANAs, APL antibodies, and ACL antibodies and their relationship with severity (Table 6).

**Table 5:** Association between autoantibodies of IgE class and the severity of atopic dermatitis

References	Study design and size	Diagnostic criteria for eczema	Measure of severity	Autoreactivity measurement methods	Results
Altrichter et al. [18]	*Case series* 192 patients with eczema (All adults. Patients with eczema ranged from 18 to 80 years, with a mean age of 39 years. Control subjects ranged from 25 to 64 years, with a mean age of 31 years. Insufficient data to calculate SD.)	*Clear* Hanifin and Rajka	*IGA Score* *Eczema Area and Severity Index*	*Western blot* Serum IgE against epithelial cell line A431 + epidermis	‘Disease activity…was also *significantly higher* in autoreactive patients than in non-autoreactive patients’ *(P* < *0.001)*
Hide et al. [47]	Case–control study* 66 patients with eczema (17 mild, 20 moderate, 26 severe and 3 undefined) (Patients with eczema ranged from 13 to 37 years, with a mean age of 24.7 ± 5.2 years.)	*Moderately clear* Severity based on grading by Rajka and Langeland	*Rajka and Langeland*	*Skin test* Intradermal injection of autologous sweat	*No association* between frequency of reaction to autologous sweat and severity of AD No statistical analysis shown Mild: 82.4% (14/17) Moderate: 90.0% (18/20) Severe: 80.8% (21/26) Undefined: 100% (3/3)
Hiragun et al. [20]	Case–control study* 63 patients with AD (Patients with AD ranged from 0 to 65 years, with a mean age of 29.9 ± 11.5 years.)	*Moderately clear* ‘severity of AD was evaluated using severity index of Japanese guideline for AD’	*Severity index of Japanese guidelines for AD*	*ELISA* Specific to QRX and rMGL_1304	*Spearman’s Correlation Coefficients* QRX-specific IgE *R* = *0.5468, P* < *0.0001* rMGL_1304-specific IgE: *R* = *0.448, p* < *0.0001*
Ilves, Virolainen and Harvima [21]	Case–control study* 50 patients with AD (Patients with AD ranged from 21 to 64 years, with a mean age of 38 years. Insufficient data to calculate SD.)	*Clear* Rajka and Langeland	*Own definition* 1 = symptomless 2 = almost symptomless 3 = mild eczema 4 = moderate eczema	*Intracutaneous injection* Of autologous sweat	*Linear trend in Chi-squared test* *(P* = *0.009)* *Moderately positive correlation* between disease severity and positive intracutaneous test *(*rs = *0.37, p* = *0.008)*
Kinaciyan et al. [48]	*Case report* 1 patient with eczema (The patient was 26 years)	*Not clear*	*SCORAD*	*Western blot* Whole serum IgE against human epithelial cell extracts	‘association between severity of skin manifestations and IgE autoreactivity’
Kohsaka et al. [49]	*Case series* 18 patients with mild AD 23 patients with moderate AD 12 patients with severe AD 11 patients with most severe AD (Patients with AD had a mean age of 28.2 ± 11.3. years.)	*Clear* Japanese Dermatological Association (JDA) criteria for AD	*Severity index of Japanese guidelines for AD*	*Western blot* Levels of IgE binding against MGL_1304 and its homologs	*Spearman’s Correlation coefficient significant for all tested antigens* TF-rMGL_1304 *r* = 0.3995, *P* < 0.005 TF-rMala s 8 *r* = 0.3094, *P* < 0.05 TF-rMala r 8 *r* = 0.3588, *P* < 0.005
Lucae *et al*. [50]	*Case reports* 4 Patients with AD underwent treatment with Cyclosporine C (All adults. Patients with AD ranged from 31 to 54 years. Insufficient data to calculate mean and SD.)	*Clear* Hanifin and Rajka	*SCORAD*	*Western blot* IgE against Human Epithelial Cell Extracts	‘The intensity of IgE autoreactivity…seemed to reflect skin inflammation’
Mittermann *et al*. (2016) [25]	Case–control study* 179 patients with AD (53 severe + 126 moderate) (All adults. Patients ranged from 18 to 65 years, with a median age of 28 years. Insufficient data to calculate mean and SD.)	*Clear* UK Working Party’s Diagnostic Criteria for Atopic Dermatitis	*SCORAD*	Immunoblot assay Specific to human A431 extract	*30% of patients with severe AD* displayed autoreactivity compared to *13% of patients with moderate AD* *Significant Association (P* < *0.01)*
Mothes et al. [26]	Case–control study* 174 patients with eczema (All adults. Eczema patients had a mean age of 35.4 ± 14.8 years. Control subjects had a mean age of 36.5 ± 16.5 years.)	*Clear* Hanifin and Rajka	*Diepgen score*	*Western blot* IgE reactivity to a variety of human epithelial antigens	Significantly higher total Diepgen score *(P* = *0.036) in AA* + vs *AA- patients*
Natter *et al*. (1998) [27]	Case series 51 patients with eczema (14 children and 37 adults. Patients with eczema ranged from 1 to 63 years, with a mean age of 26.9 ± 18.6 years)	*Clear* Hanifin and Rajka	*Own score*	*Western blot* Whole serum IgE against endothelial cells and A431	12/12 patients with ‘intensive IgE reactivity to human proteins’ had severe or moderate AD 16/29 patients without IgE autoreactivity displayed severe or moderate AD
Schmid-Grendelmeier et al. [28]	Case–control study* 69 patients with eczema (All adults. Patients with eczema had a mean age of 29.3 ± 5.6 years. Other participants had a mean age of 25.3 years, but insufficient data to calculate SD)	*Clear* Hanifin and Rajka	*SCORAD*	**ELISA** Allergen-specific against rhMnSOD	**Correlation between SCORAD and anti-human MnSOD IgE level** *R* = 0.756 *P* < *0.0001*
Tanaka et al. [51]	Case–control study* 61 patients with AD (Patients with AD ranged from 2 to 43 years, with a mean age of 24.0 ± 7.5 years)	*Moderately clear* Severity based on grading by Rajka and Langeland	*Rajka and Langeland*	*Histamine release* By basophils upon stimulation by semi-purified sweat antigen	‘The extents of histamine release were *not statistically correlated* with disease severity’
Zeller et al. [30]	Case–control study* 71 patients with AE (Patients with AE had a mean age of 33.35 ± 12.70 years. Patients with non-atopic AE had a mean age of 39.35 ± 13.5 years.)	*Clear* Hanifin and Rajka and European Academy of Allergology and Clinical Immunology	*SCORAD*	*Western blot and ELISA* Against a variety of self-antigens	No significant difference in SCORAD between patients with and without IgE against self-antigens *(P* < *0.7625)*

**Table 6:** Association of autoantibodies with severity of atopic dermatitis

Marker of autoimmunity	Papers that indicate a link between AD disease severity and autoimmunity	Papers that do not indicate a link between AD disease severity and autoimmunity
IgE autoantibodies	**10** Altrichter et al. [18]* Hiragun et al. [20]* Ilves, Virolainen and Harvima [21]* Kinaciyan et al. [48] Kohsaka et al. [49]* Lucae et al. [50] Mittermann et al. [25]* Mothes et al. [26]* Natter et al. [27] Schmid-Grendelmeier et al. [28]*	**3** Hide et al. [47] Tanaka et al. [51]^**†**^ Zeller et al. [30]^**†**^
ANAs	**0**	**2** Higashi et al. [39]^**†**^ Ress et al. [43]^**†**^
Multiple indicators	**0**	**1** Szakos et al., 2004 [34]

*Differences were statistically significant

**†**Differences were not statistically significant

Two main methods of assessing severity were used, SCORAD [44] and Rajka and Langeland [45] scores, although some researchers used other measures of severity. SCORAD was developed as a standardised method to assess severity of AD that could be used in outpatient clinics. It relies on three components: intensity (responsible for 60% of the total score), extent and subjective symptoms (each responsible ≈ 20% of the score). Rajka and Langeland also put forward a simple scoring index for AD using three parameters, each scored out of 3: extent, course (how much remission in a year) and intensity (scored according to how much it impacts sleep). The sum of these scores is calculated, with 3–4 being defined as mild, 4.5–7.5 being moderate, and 8–9 being severe.

It is difficult to produce pooled data for the studies assessing a link between IgE autoantibodies and severity of AD due to wide variation in the measures of severity and outcome measures but, as shown in Table 6; the weight of evidence indicates that there is an association between the frequency of IgE autoantibodies and disease severity in AD. Furthermore, two studies [48, 50] looked at individual patients over time, assessed levels of IgE autoantibodies and SCORAD throughout treatment, and established a temporal link between SCORAD and IgE autoantibodies. Whilst it is impossible to determine from these studies whether the decrease in IgE autoreactivity as severity of disease declines is a separate response to the cyclosporine A treatment, an unrelated biomarker resulting from a decrease in disease severity, or indeed the cause of the reduced severity, it provides strong support for a link between AD disease severity and autoimmunity. This association did not seem to be present with respect to ANAs, as neither study that looked exclusively at ANAs demonstrated a significant association (Table 6), but more studies are required to reach a definitive conclusion. Szakos et al. [34] also indicated that there was no significant difference in disease severity in patients that displayed ANAs, ACL, APL or a combination of ANAs and APL (Table 6). Overall, the available evidence indicates that severity of AD is associated with autoimmunity, but that this response might be limited to IgE-mediated autoreactivity. However, further research is required into other indicators of autoimmunity, such as IgG autoantibodies, ANAs and IgA autoantibodies to confirm this, as current evidence is limited in these areas.

## Discussion

The weight of evidence presented in this review indicates that there is an association between autoreactivity and AD, as well as disease severity in AD, particularly with regard to autoantibodies of IgE class. However, this review cannot provide evidence for a causal link between the two, as the exact mechanisms for how autoimmunity occurs in patients with AD are unclear. Some papers hypothesise that cross reactivity between *Malassezia* spp. and the autoantigen hMnSOD could underlie the pathogenic response seen in AD [21, 25], whilst many others have identified cells in the skin as a source of autoantigens, potentially indicating that skin damage could lead to an autoimmune response to components within the skin, giving rise to autoimmunity. However, these hypotheses rest on assumptions that have not been proven. Taking all the available evidence into account, it is not possible to declare that autoimmunity is a pathogenic factor underlying the natural history of AD, but it does seem plausible and certainly worthy of future research.

This review points to several areas, where future research into autoimmunity and AD would be most valuable. First, some areas of autoimmunity have been less thoroughly investigated when looking at an association with AD. Whilst IgE autoantibodies and, to some extent, ANAs have been covered extensively by research, other markers of autoimmunity have less evidence available. Studies investigating IgG autoantibodies, for example, were varied in terms of control groups, often comparing against groups that have other diseases, and were less clear about the selection criteria for the patients with AD. More research into IgG autoantibodies in AD, across a wide range of autoantigens and in comparison to healthy control groups, would be useful in drawing more robust conclusions.

Five other studies, not specifically covered by this review, have examined aspects of autoimmunity other than autoantibodies (Table 7). Collectively, these studies do indicate that autoimmunity is related to AD, with three of the five indicating statistically significantly higher rates of autoimmune response in patients with AD compared to their control groups, be it through increased histamine release upon stimulation by semi-purified sweat antigens [51], activation of iNKT cells without the corresponding ligand [52], or significantly higher stimulation index of PBMCs in response to hsp60 [53]. In addition, Hide et al. [47] show a greater frequency of response to intradermal injection of autologous sweat in AD patients, albeit without statistical analysis. The final study [54] does not seem to indicate an increased autoreactive T-cell response in patients with AD, but does indicate that there may be an alteration to the balance of CLA+/CLA− and CCR4+/CCR4− cells. Overall, these less traditional indicators of autoimmunity show promising results in terms of a link with AD, but further research is required to come to definitive conclusions.

**Table 7:** Association between markers of autoimmunity (other than autoantibodies) and atopic dermatitis

References	Study design and size	Diagnostic criteria for AD	Methods	Prevalence of AR in control subjects	Prevalence of AR in patients with AD
Heratizadeh et al. [54]	Case–control study 30 adult patients with eczema 12 healthy individuals 3 non-atopic patients with chronic plaque psoriasis (Patients with eczema ranged from 18 to 65 years. Insufficient data to calculate mean and SD. No information given about control subjects.)	*Clear* Hanifin and Rajka	Analysis of peripheral blood mononuclear cells via proliferation rate of blood-derived lymphocytes in response to α-NAC	Median: *9.3%* with α-NAC, *0.8%* with medium control	Median: *8.4%* with α-NAC, *1.5%* with medium control (both statistically significantly increased with α-NAC compared to control medium (*P* < 0.001), no analysis compared to control group)
Hide et al. [47] Skin Patch Test	Case–control study 66 patients with eczema 7 patients with allergic rhinitis 27 healthy control patients (Patients with AD ranged from 13 to 37 years, with a mean age of 24.7 ± 5.2 years. Patients with allergic rhinitis ranged from 20 to 35 years, with a mean age of 24.6 ± 4.8 years. Control subjects ranged from 20 to 42 years, with a mean age of 27.8 ± 6.7 years.)	*Moderately clear* Severity based on grading by Rajka and Langeland	*Intradermal* *injection* of autologous sweat	*11.1%* 3/27 control patients *71.4%* 5/7 patients with allergic rhinitis	*85%* 56/66 eczema patients
Kapitein et al. [53]	Case–control study 28 child patients with AD (of 55) 18 healthy control children (of 30) (Patients with AD ranged from 1.5 to 17.5 years, with a mean age of 8.8 years. Healthy controls ranged from 1.2 to 17.3 years, with a mean age of 8.6 years. Insufficient data to calculate SD.)	*Clear* Hanifin and Rajka	*Stimulation index* of peripheral blood mononuclear cells in response to hsp60	–	*‘Significantly higher’* *(P* = *0.004)*
Lind et al. [52] IL-18 and iNKT cells	Case–control study 78 patients with eczema 45 healthy subjects (Patients with eczema ranged from 18 to 65 years, with a mean age of 29 years. Healthy controls ranged from 20 to 64 years with a mean age of 39 years. Insufficient data to calculate SD.)	*Clear* UK Working Party’s Diagnostic Criteria for Atopic Dermatitis	*Plasma IL-18 levels and iNKT autoreactivity* 2 part hypothesis that IL-18 can activate iNKT cells ‘independently of exogenous ligands’ and that IL-18 correlates with AE disease severity	kawamom-	*Levels of IL-18 significantly higher (P* = *0.003) in AE patients than healthy controls*
Tanaka et al. [51] Histamine release	Case–control study 61 patients with AD 13 patients with psoriasis 46 healthy control patients (Patients with AD ranged from 2 to 43 years, with a mean age of 24.0 ± 7.5 years. Patients with psoriasis ranged from 29 to 73 years, with a mean age of 53.4 ± 17 years. Healthy controls ranged from 1 to 52 years, with a mean age of 28.4 ± 8.9 years.)	*Moderately Clear* Severity based on grading by Rajka and Langeland	Measurement of *histamine released* by basophils upon stimulation by *semi-purified sweat antigen*	*8.7%* 4/46 healthy control subjects *0%* 0/13 patients with psoriasis	*77%* 47/61 patients with AD *(P* < *0.001 compared to healthy controls)*

There are also new indicators of autoimmunity that, whilst not meeting the inclusion criteria of this review, could provide insight into AD. Some interesting papers have investigated the function of regulatory T cells (Tregs) in AD with research indicating that the suppressive effect of Tregs on CD8^+^CLA^+^ T-cell proliferation is inhibited in AD [55]. This would be a similar mechanism to other autoimmune/inflammatory diseases such as psoriasis, which shows impaired suppression of CD4^+^CD25^−^ T cells [56]. Zhang et al. [55] note that further experiments are needed to address the theory that the CD8^+^CLA^+^ T cells in patients with AD might resist suppression, as opposed to there being dysregulation of Tregs, but further research into this field could provide a valuable insight into the mechanism behind autoimmunity. Later research corroborated this [57], with both papers suggesting that reduced inhibition of Teff cells could cause impaired self-tolerance in patients with AD, but further research would assist in elucidating specific mechanisms underlying the autoimmune response.

Another area that could provide useful research into autoimmunity and AD is analysis of genetic variants and potential associations with AD. For example, copy-number variations of the human histamine H4 receptor (HRH4) gene have been shown to be associated with AD [58], as well as a number of other autoimmune conditions such as SLE [59]. Whilst the exact mechanisms involved with the human histamine H4 receptor gene and AD are unclear, Chen et al. [58] suggest that the expression of HRH4 could be enhanced because of inflammatory stimuli found in autoimmune diseases, leading to a stronger response to histamine. This is an example of another potential component to the autoimmune pathogenesis in AD and is an area that warrants future research.

How autoimmunity is related to AD in animal models also warrants further research. Using animal models could lead to greater understanding of whether autoimmunity causes AD, perpetuates AD, or whether it is just an associated factor. Differences in ethical considerations for humans and animals allows for different studies to be carried out in animals, allowing more flexibility in study design and an ability to more easily identify causation.

Finally, further research into how different phenotypes of AD are associated with autoimmunity could provide a strong basis for future clinical research. Patients with AD can be divided into intrinsic and extrinsic categories, as well as other groups, including age of diagnosis and ethnic origin [60]. These groups have different disease characteristics, so more research into the underlying pathogenic mechanisms within these groups, be it related to autoimmunity or other factors, would be useful to aid understanding and potentially improve future treatments.
